# Supplementary figures and images for: Identification of two CiGADs from Caragana intermedia and their transcriptional responses to abiotic stresses and exogenous abscisic acid
Source: PeerJ. 2017 Jun 14;5:e3439. doi: 10.7717/peerj.3439 (PMC5473354; doi:10.7717/peerj.3439)

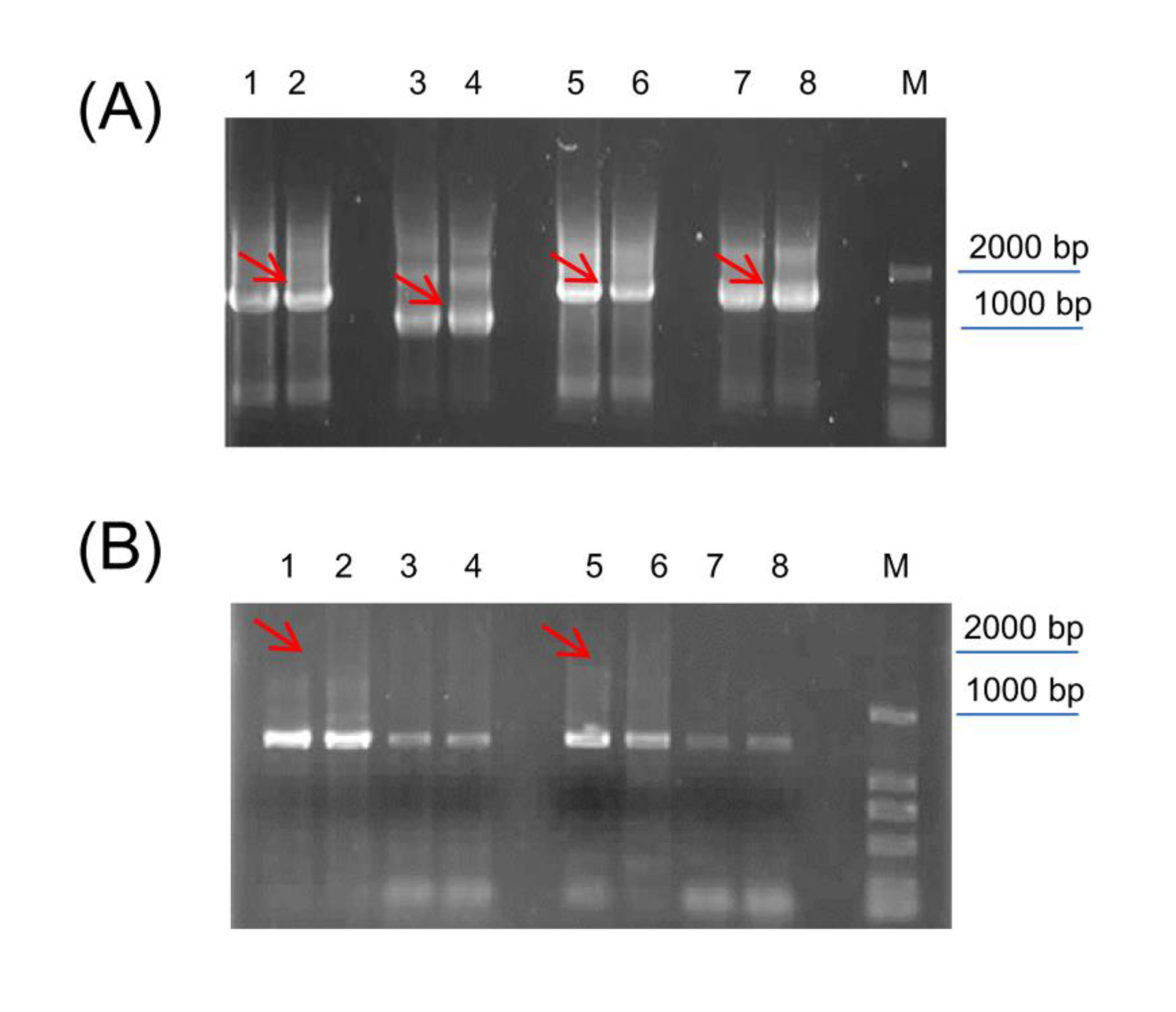

Supplement: Figure S1 — (A) Lane 1, 2: 5′ RACE of CiGAD1; Lane 3, 4: 5′ RACE of CiGAD2; Lane 5, 6: 3′ RACE of CiGAD1; Lane 7, 8: 3′ RACE of CiGAD2; the sequences included the common primer of UPM. (B) Lane 1, 2, 3, 4: PCR amplification of open reading frame (ORF) of CiGAD1; Lane 5, 6, 7, 8: PCR amplification of ORF in CiGAD2. Red arrows were targeted bands; M: 2,000 bp marker. [file peerj-05-3439-s001.png]

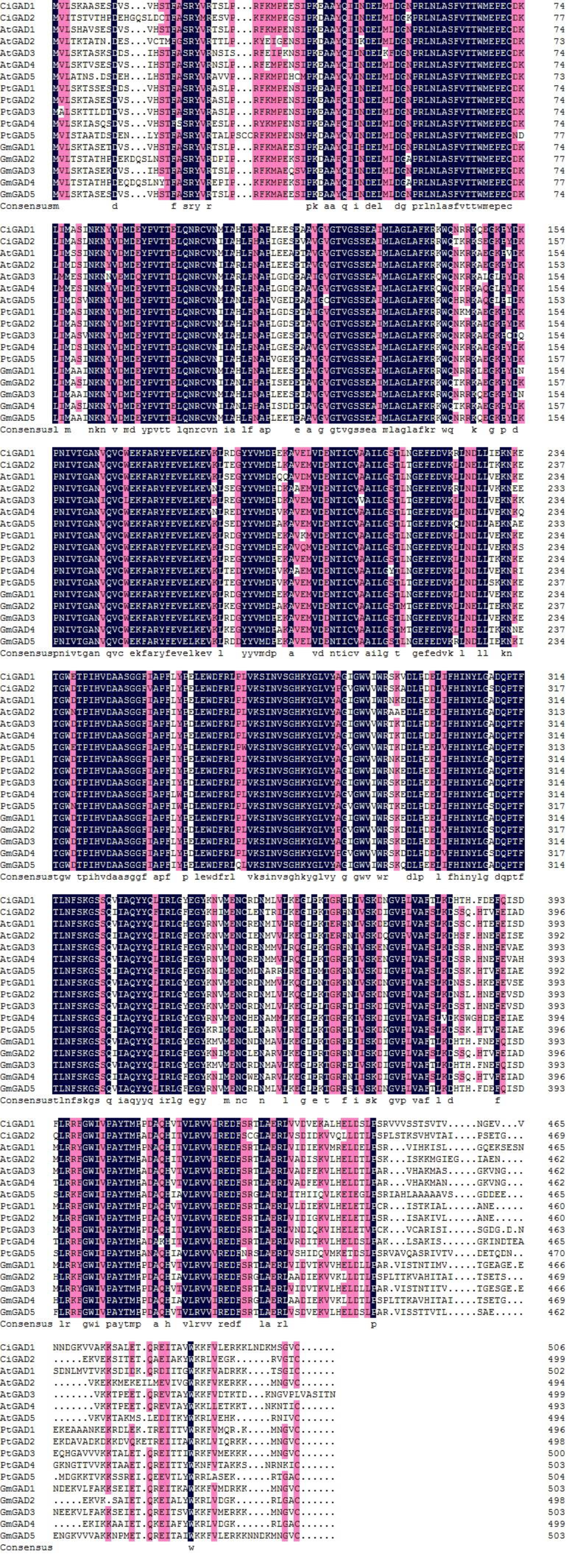

Supplement: Figure S2 — Accession number: AtGAD1 (AT5G17330), AtGAD2 (AT1G65960). , AtGAD3 (AT2G02000), AtGAD4 (AT2G02010), AtGAD5 (AT3G17760), PtGAD1 (Potri.T059200), , PtGAD2 (Potri.004G075200), PtGAD3 (Potri.004G075300), PtGAD4 (Potri.010G100500),, PtGAD5 (Potri.012G039000), , GmGAD1 (Glyma02g40840), , GmGAD2 (Glyma09g29900), GmGAD3 (Glyma14g39170), GmGAD4 (Glyma16g34450), and GmGAD5 (Glyma18g04940). The abbreviation of gene names are as follows: At, Arabidopsis thaliana; Pt, Populus trichocarpa; Gm, Glycine max. [file peerj-05-3439-s002.png]
