# Supplementary material for: Identification of two CiGADs from Caragana intermedia and their transcriptional responses to abiotic stresses and exogenous abscisic acid
Source: PeerJ. 2017 Jun 14;5:e3439. doi: 10.7717/peerj.3439 (PMC5473354; doi:10.7717/peerj.3439)
Supplement: Table S1 [file peerj-05-3439-s003.pdf]

	Primer name	Primer sequences
5'- RACE	*CiGAD1*-1	AGTGCCDGCDTACDCCDTGC
	CiGAD1-2	CGGAGCTGACCDACCCDCCTTTACCC
3'- RACE	*CiGAD1*-3	ACDAACDCCDCTCDTCTTATC
	CiGAD1-4	CCDCCCDAAGCGCCTCDAAAAATCDG
5'- RACE	*CiGAD2*-1	ATGCDCDACDCDTCDCTGTTC
	CiGAD2-2	GGGGCTAATGTGCDGGTTTGTTGGGA
3'- RACE	*CiGAD2*-3	GCDTGTTCCDACTCTCTTG
	CiGAD2-4	GCCDAGCCGCDACTGAAATCCTCCCT
Full-length	*CiGAD1*-5F	GATGGTTCTCTCCAAGGCCGCCTC
	CiGAD1-5R	CTAACAAACACCACTCATCTTA CC
	CiGAD2-5F	G ATGGTTCTCTCCAAGGCCGCCT
	CiGAD2-5R	AACAAACACCACTCTCATCTTA
