# Supplementary material for: Identification of two CiGADs from Caragana intermedia and their transcriptional responses to abiotic stresses and exogenous abscisic acid
Source: PeerJ. 2017 Jun 14;5:e3439. doi: 10.7717/peerj.3439 (PMC5473354; doi:10.7717/peerj.3439)
Supplement: Table S2 [file peerj-05-3439-s004.pdf]

Gene name	Primer name	Primer sequences
EF1α	*Act-F*	TGGGTGGGACATTCTCTGATT
	Act-R	GCACGGTTCACTTCTTCTTAGC
CiGAD1	*1F-1*	CCATGCCTCCAGATGCTCAA
	1R-1	ACTTCTTCCAAACCGCAGTGA
	1F-2	ACCGCAGTGATTTCCCTTTGA
	1R-2	TACACCATGCCTCCAGATGC
CiGAD2	*2F-1*	ACAAGTCTCTCAGCCAAGCC
	2R-1	GGAGTGCCACTTGTAGCCTT
	2F-2	CTCCTCCAGACAACCCAACC
	2R-2	TGTAGATGCTGCAAGTGGGG
